# Extremely Late Diagnosis of Hereditary Angioedema Type I in an Elderly Female

**DOI:** 10.7759/cureus.57303

**Published:** 2024-03-31

**Authors:** Jonathan Estaris, Marina Ostroukhova

**Affiliations:** 1 Medicine, University of Hawaiʻi at Mānoa, Honolulu, USA; 2 Allergy and Immunology, Aloha Allergy and Immunology, Honolulu, USA

**Keywords:** hae, hae with low c1 inhibitor, delayed diagnosis, c1 esterase inhibitor, angioedema, hae type i, hereditary angioedema without normal c1 inhibitor, hereditary angioedema

## Abstract

This case presents an instance of an extremely delayed diagnosis of hereditary angioedema (HAE) type I in an elderly female with no significant past medical history. The patient had a prolonged history of recurrent lip swelling and itchiness dating back to her teenage years, leading to multiple visits to the emergency room (ER). These recurrent episodes were characterized by random onset and accompanied by generalized pruritus and urticaria. During these ER visits, the patient would be inappropriately treated for presumed hypersensitivity reaction due to her confounding environmental allergies presenting with urticaria, complicating and significantly delaying her diagnosis. The patient was adopted, and the family history was unknown. There was no history of medication use suggestive of acquired angioedema. At the time of the visit, she had signs of chronic lip changes and atopy. After an extensive workup, it showed severely low levels of C1 esterase inhibitor and borderline low to normal C4 and C1q, consistent with the diagnosis of HAE type I. Initial treatment with an on-demand C1 esterase inhibitor reduced the recurrence of lip swelling and transitioned to long-term prophylaxis use. Overall, the treatment outcome was generally successful, with less recurrence of lip swelling and ER visits.

## Introduction

Hereditary angioedema (HAE) is a relatively rare disease that presents with recurrent episodes of angioedema of the skin and mucosal tissue, particularly of the upper respiratory and gastrointestinal tracts. Although the swelling is self-limited and resolves in two to five days without treatment, laryngeal involvement may cause fatal asphyxiation [1]. The diagnosis of HAE is typically made during childhood and adolescence. However, considerable delays in diagnosis are reported, with a median delay of 8.5 years [2], which burdens their quality of life considerably, leading to unnecessary treatments. Due to the low prevalence and unspecific symptoms, the final diagnosis is often made after a long delay [3]. The few reported delayed diagnoses into adulthood are often acquired secondary to underlying or inciting disease or type II characterized by normal C1 esterase inhibitor level but decreased function [4]. Here, we present an interesting case of an extremely delayed case of HAE type I in an elderly female.

## Case presentation

The patient was a 66-year-old female with no significant medical history. She presented in the clinic endorsing a long-standing history of multiple ER visits dating back to her teenage years, associated with recurrent, random episodes of lip swelling and itchiness, along with generalized pruritus and urticaria. These episodes occur sporadically, approximately once every two to three years. During more severe presentations, she would seek acute visits in the ED where she would be treated with antihistamines and topical steroids for presumed hypersensitivity reaction. Her symptoms would generally self-resolve on their own. These episodes would continue infrequently for several decades but become more recurrent in the past year. Her symptoms were generally self-limiting, including reports of occasional periorbital and lip puffiness, intermittent shortness of breath with palpitations, and mild abdominal pain. Ultimately, she was referred to an allergist for an extensive workup of her recurrent lip swelling and urticaria.

At the time of the initial clinic visit, she presented with signs of chronic lip changes, which were very dry, scaly, and fissured under a hyperpigmented erythematous base. She had a prominent nasal crease and congested nasal turbinates on direct rhinoscopy. The skin exam was positive for a few urticarial rashes on her extremities. Further allergic and environmental history did not reveal an association with food or exposure to specific medication, chemicals, or environmental allergens. The patient was not taking any chronic medication, such as angiotensin-converting enzyme (ACE) inhibitors or nonsteroidal anti-inflammatory drugs (NSAIDs). She worked in an animal sanctuary and was exposed to reptiles, cats, and dogs. Of note, she was adopted. Thus, family history was unknown. Skin itchiness was reported to be more pronounced with spicy foods and exposure to volcanic smog. An initial allergy skin testing was unrevealing and was only positive for a few environmental allergens, including dust mites and tree pollen. Given her unremarkable allergy sensitivities, which were believed to have chronically masqueraded her late diagnosis and without apparent triggers, an extensive workup for angioedema was performed.

Conducting a comprehensive workup for angioedema is crucial for establishing the diagnosis in this case. Although she presented with urticaria, given her long history of recurrent angioedema without significant improvement with typical medications, broad differentials were considered to differentiate mast-cell-mediated (histaminergic) and bradykinin-induced etiologies of angioedema.

Levels of C1 esterase inhibitor (functional and quantitative), C1q complement, and C4 complement were pivotal in diagnosing HAE. We also performed allergic skin testing for environmental allergies, given the reported history. In addition, we obtained the total immunoglobulin E level to ascertain allergic disease and tryptase level to rule out mast-cell-mediated disease. We also performed baseline complete blood count (CBC), comprehensive metabolic panel (CMP), C-reactive protein (CRP), erythrocyte sedimentation rate (ESR), thyroid-stimulating hormone (TSH), anti-thyroid peroxidase (anti-TPO) microsomal, anti-nuclear antibody titer, and *Helicobacter pylori* panel to ascertain nonallergic and acquired etiologies, that is, infectious, endocrinologic, rheumatologic, and other inflammatory causes. In addition, antibody screening and serum protein electrophoresis should be used to rule out hematologic and lymphoproliferative disorders causing acquired angioedema. The ACE level was obtained to rule out confounding ACE-induced angioedema.

The following diagnostics, including TSH, ACE, anti-nuclear antibody, anti-TPO microsomal, H. pylori panel, tryptase, immunoglobulin E, and antibody screen, were unrevealing except for severely low levels of C1 esterase inhibitor (5 mg/dL, normal range 29-31 mg/dL) and borderline low-normal C4 and C1q, as seen in Table 1. These findings were consistent with a diagnosis of HAE type I.

**Table 1:** Diagnostic workup for hereditary angioedema. Abs Imm Granulo, absolute immature granulocytes; eGFR, estimated glomerular filtration rate; CKD-EPI, Chronic Kidney Disease Epidemiology Collaboration; SGOT, serum glutamic-oxaloacetic transaminase; AST, aspartate aminotransferase; SGPT, serum glutamic-pyruvic transaminase; ALT, alanine aminotransferase; ELP, electrophoresis; MCHC, mean corpuscular hemoglobin concentration; MCV, mean corpuscular volume; MCH, mean corpuscular hemoglobin

Parameter	Result	Reference range
C1 esterase inhibitor, Quant	5	21-39 mg/dL
C1q complement	5.6	5.0-8.6 mg/dL
C1 esterase inhibitor, functional	>100	>=68 Normal
C4 complement	16	15-40 mg/dL
CBC W/diff and platelet count		
White blood count	4.96	3.80 x 10^3^ to 10.80 x 10^3^/µL
Red blood cell count	4.04	3.60 x10^6^ to 5.40 x 10^6^/µL
Hemoglobin	12.1	11.2-15.7 g/dL
Hematocrit	37.1	34.1%-44.9%
MCV	91.8	79.4-98.4 fL
MCH	30	26.0-34.0 pg
MCHC	32.6	32.0-36.0 g/dL
RDW	12.8	11.6%-14.4%
Platelet count	288	151 x 10^3^ to 424 x 10^3^/µL
Imm granulocyte	0.2	0.0%-1.0%
Neutrophil	60.6	34.0%-72.0%
Lymphocyte	30.2	12.0%-44.0%
Monocyte	5.6	0.0%-12.0%
Eosinophil	2.8	0.0%-7.0%
Basophil	0.6	0.0%-2.0%
Abs Imm Granulo	0.01	0.0 x 10^3^ to 0.10 x 10^3^/µL
Abs neutrophils	3	1.56 x 10^3^ to 6.20 x 10^3^/µL
Abs lymphocytes	1.5	1.18 x 10^3^ to 3.74 x 10^3^/µL
Sedimentation rate	26	0-30 mm/hour
Antibody screening	Negative	Negative
TSH	1.22	0.27-4.20 uIU/mL
Immunoglobulin E	60.4	<100 IU/mL
C-reactive protein (CRP)	<0.6	0.0-10.0 mg/L
Serum protein ELP w/interpretation		
Total protein	7.7	6.4-8.3 g/dL
Total protein	7.7	6.4-8.3 g/dL
Albumin	4.78	3.80-5.20 g/dL
Alpha 1	0.22	0.10-0.25 g/dL
Alpha 2	0.8	0.50-1.05 g/dL
Beta	0.76	0.50-1.03 g/dL
Gamma	1.13	0.51-1.47 g/dL
Comprehensive metabolic profile		
Glucose	101	70-99 mg/dL
BUN	14	6-23 mg/dL
Creatinine	0.7	0.6-1.4 mg/dL
eGFR, CKD-EPI, non-African AM	93	>=90* mL/minute/1.73 m^2^
eGFR, CKD-EPI, African AM	108	>=90* mL/minute/1.73 m^2^
Sodium	139	133-145 mEq/L
Potassium	4.2	3.3-5.1 mEq/L
Chloride	100	95-108 mEq/L
CO_2_	24	21-30 mEq/L
Calcium	9.6	8.3-10.5 mg/dL
SGOT (AST)	27	0-40 IU/L
SGPT (ALT)	34	0-41 IU/L
Alkaline phosphatase	71	35-129 IU/L
Total bilirubin	0.3	0-1.2 mg/dL
Total protein	7.7	6.4-8.3 g/dL
Albumin	5.1	3.5-5.2 g/dL
Globulin	2.6	2.1-3.7 g/dL
Albumin/globulin ratio	2	1.0-2.2
Anti-nuclear Ab titer	<40	=<40
H. pylori antibody, IgG	Negative	
H. pylori antibody, IgA	Negative	
Anti-TPO microsomal	<15	<34 IU/mL
Tryptase	3	<11.0 µg/L
Angiotensin-converting enzyme	50	9-67 U/L

Ultimately, treatment with a C1 esterase inhibitor, with on-demand usage on top of long-term prophylaxis, led to significant improvement and prevention of attacks and ER visits. In addition, environmental allergies were controlled by antihistamines and allergen avoidance.

## Discussion

The diagnosis of HAE is challenging and notoriously leads to significant delays in diagnosis. Early recognition and high suspicion of HAE are vital in avoiding catastrophic and fatal outcomes. Traditional teaching suggests that HAE presents without urticaria, which becomes a common pitfall, misdiagnosing these cases as hypersensitivity reactions, which leads to inappropriate treatment with antihistamines or steroids, such as in this case. More often, the lack of awareness about rare conditions or possible anchoring bias further delays the diagnosis of HAE. In 2013, Zanichelli et al. reported that the median delay in diagnosis of HAE type I and II was 8.5 years in Europe [2]. The median age at first symptoms was 12.0 years, and the corresponding median age at diagnosis was 24.3 years [5]. The disease is frequently misdiagnosed and mistreated, leading to several unnecessary procedures that may potentially mask severe life-threatening complications such as laryngeal edema. Although more commonly, the diagnosis is made during childhood and adolescence; late diagnoses can also be made in adulthood. Cases of extremely late diagnosis HAE were reported by Berger et al. [4], wherein HAE type 2, with normal C1 esterase inhibitor but decreased function, was diagnosed at the age of 80 after multiple tests for abdominal pain revealed unremarkable results. In our case, the delay in the diagnosis was complicated by the delay in recognition and suspicion of HAE due to concomitant chronic urticaria. Based on the International World Allergy Organization/European Academy of Allergy and Clinical Immunology (WAO/EAACI) guideline in 2017, HAE is suspected if the patient presents with a history of recurrent angioedema attacks plus the absence of urticaria (wheals) among other concomitant signs and symptoms. Although pruritic urticaria usually makes the diagnosis of HAE unlikely [6], the occurrence of urticaria does not necessarily exclude HAE [7]. In our case, the patient's concomitant allergic sensitivities chronically masqueraded and significantly delayed the diagnosis of HAE type I. 

Type I HAE is the most common subtype involving a mutation in the C1-INH gene, *SERPING1*. This results in truncated or misfolded proteins, which are subsequently not secreted, leading to low C1-INH levels. Type II HAE results in a normal C1-INH level but defective function. Finally, the third type is described with normal C1-INH levels, undistinguishable between type I and type II, postulated to be due to either an increased activity of factor XII or congenital deficiency of enzymes such as ACE, carboxypeptidase N, and α2-macroglobulin or a phenotypic decrease in the function of these enzymes [8].

A multidisciplinary approach to prevent HAE attacks is critical in treating and managing HAE, including avoiding triggers and availing of on-demand therapies. Options for on-demand therapy include plasma-derived C1-INH concentrate, recombinant C1-INH concentrate, icatibant, and ecallantide. Regular plasma-derived C1 inhibitor concentrate injections are effective and well tolerated and generally achieve long-term prophylaxis [5]. 

Treatments available for HAE depend on the etiology of the Angioedema. Given the pathophysiology of HAE type I, wherein low levels of C1 esterase inhibitor, select FDA-targeted therapies are available. Due to availability, the patient was started with a recombinant C1 esterase inhibitor as an on-demand treatment. This has been shown to prevent the recurrence of angioedema needing an ER visit. However, due to the difficulty of intravenous administration, it was opted to shift to plasma-derived C1 inhibitor concentrate for long-term prophylaxis.

Genetic testing could be offered and may help differentiate HAE-C1-INH from acquired C1-INH deficiency. Prenatal testing could be considered in cases with a significant family history of HAE, or in situations where repeated biochemical C1-INH test results are ambiguous [9].

## Conclusions

HAE is a potentially fatal disease that is poorly diagnosed. Awareness of its diagnosis and maintaining a high index of suspicion are essential in preventing delays in diagnosis and avoiding inadequate and inappropriate treatment, especially in cases where symptoms may overlap with other conditions such as chronic spontaneous urticaria or other allergic diseases. As observed in our case, the patient presented with concomitant chronic urticaria related to environmental allergies, which masked the diagnosis of HAE. An important lesson in this case is that angioedema, even with urticaria, does not directly translate to a hypersensitivity reaction. Patients may have urticaria from other underlying etiologies which healthcare practitioners should recognize. It is important to have a high index of suspicion for HAE especially if they are already presenting with chronic, recurrent episodes of angioedema without clear inciting factors. Prompt referral to an allergist or a specialist is also essential to avoid unnecessary and invasive procedures to provide early lifesaving treatment.
